# Fibroepithelial Polyp in a Child: A Rare Pathology of Upper Urinary Tract Obstruction

**DOI:** 10.7759/cureus.8748

**Published:** 2020-06-21

**Authors:** Saeed Alhindi, Husain Alaradi, Mohamed Mubarak

**Affiliations:** 1 Pediatric Surgery, Salmaniya Medical Complex, Manama, BHR; 2 Surgery, Salmaniya Medical Complex, Manama, BHR

**Keywords:** upper urinary tract obstruction, fibroepithelial polyp, hydroureter, hydronephrosis

## Abstract

Fibroepithelial polyp is a rare benign tumor of the urothelial system that originates from the mesoderm. Polyps are usually small and located in the upper urinary tract and ureteropelvic junction. However, in the pediatric population, such polyps are more common in the posterior urethra and will present with symptoms of urinary tract obstruction. Some will present with flank pain and hematuria, resembling symptoms of ureteric stones. In this case, we discuss a nine-year-old boy presenting with complaints of flank pain and hematuria for one year. Following laboratory and radiological investigations, the left ureter was dilated at the mid-lumbar region with an anteroposterior diameter of 2.3 x 0.6 cm and a left renal pelvis anteroposterior diameter of 2.2 cm. An ultrasound scan identified an intraluminal lesion suspected to be a fibroepithelial polyp. Management was carried out via retroperitoneal surgery with upper ureteral resection and end-to-end anastomosis. Postoperatively, the patient’s symptoms improved, and a subsequent ultrasound scan and renal function test showed improvement of the left hydroureter and hydronephrosis.

## Introduction

Fibroepithelial polyps are rare benign tumors that can occur in the urinary tract. It is an uncommon pathology in children, as most cases are reported in adults. Furthermore, most polyps are usually small, short, and located in the upper ureter and ureteropelvic junction [1]. Diagnosis of fibroepithelial polyps can be made via ultrasonography followed by either intravenous urogram or retrograde pyelogram. Ureteroscopy has also been reported as a feasible option for diagnosis but is very difficult among young children due to the small working space and size of the polyp [2]. An array of options can be utilized to manage such cases: segmental resection with ureteroureterostomy, nephroureterectomy, laser coagulation, and ureteroscopic polypectomy [3]. We present the case of a nine-year-old boy presenting with concerns of flank pain and hematuria that turned out to be, following further investigation, a fibroepithelial polyp of the ureter causing upper urinary tract obstruction.

## Case presentation

**Figure 1:**
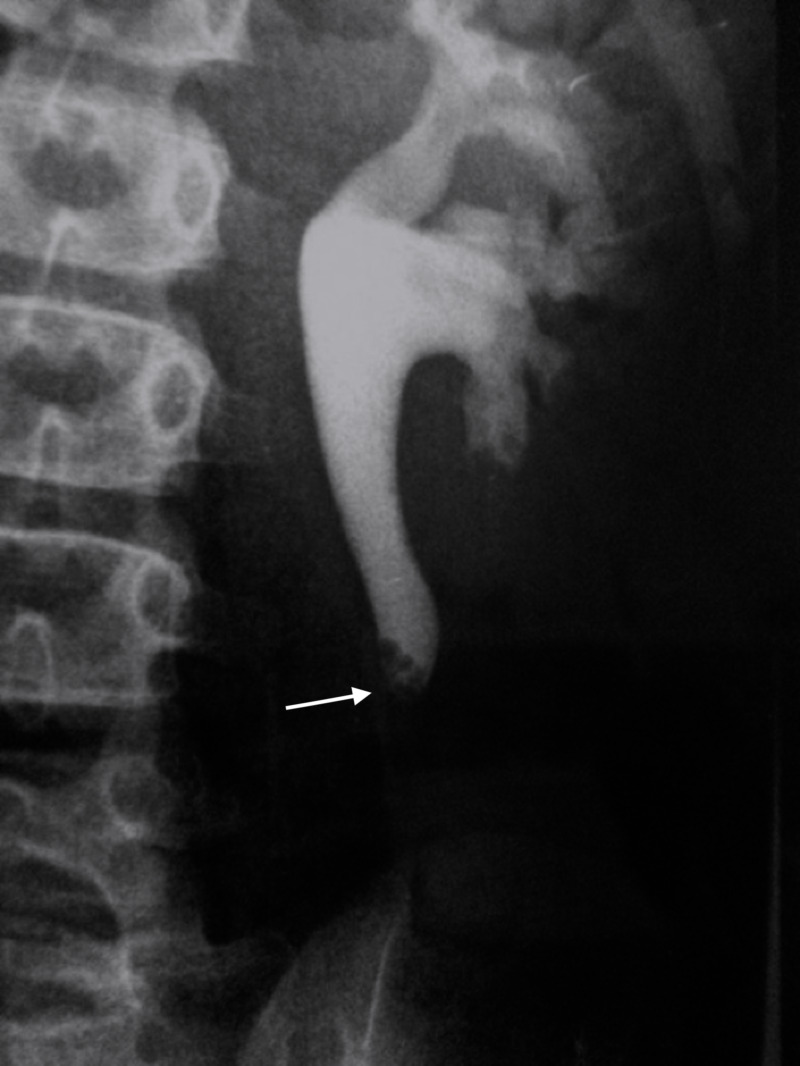
Intravenous Pyelogram of the Left Upper Ureter Intravenous pyelogram showing a left upper ureteric defect.

A nine-year-old boy presented to the clinic with a history of flank pain and hematuria for the past year. He was not known to have any medical illness and had normal developmental growth milestones. Physical examination revealed no evident abnormality. Routine laboratory workup showed a healthy complete blood count and renal function. On an ultrasound scan, left-sided hydronephrosis was seen. There was no evidence of cortical thinning, but the left ureter was dilated. This was traced to the mid-lumbar region. At that point, there was an oval-shaped isoechoic, mildly heterogeneous lesion within the ureter measuring 2.3 x 0.6 cm. This intraluminal lesion in the mid-left lumbar ureter was causing a mild hydroureter and hydronephrosis, suggestive of a fibroepithelial polyp. Assessment with color Doppler ultrasonography was difficult, but there appeared to be some internal vascularity within the lesion. The anteroposterior diameter of the left renal pelvis measured 2.2 cm. An intravenous pyelogram (IVP) was done and showed a left upper ureteric filling defect <1 cm (Figure 1).

**Figure 2:**
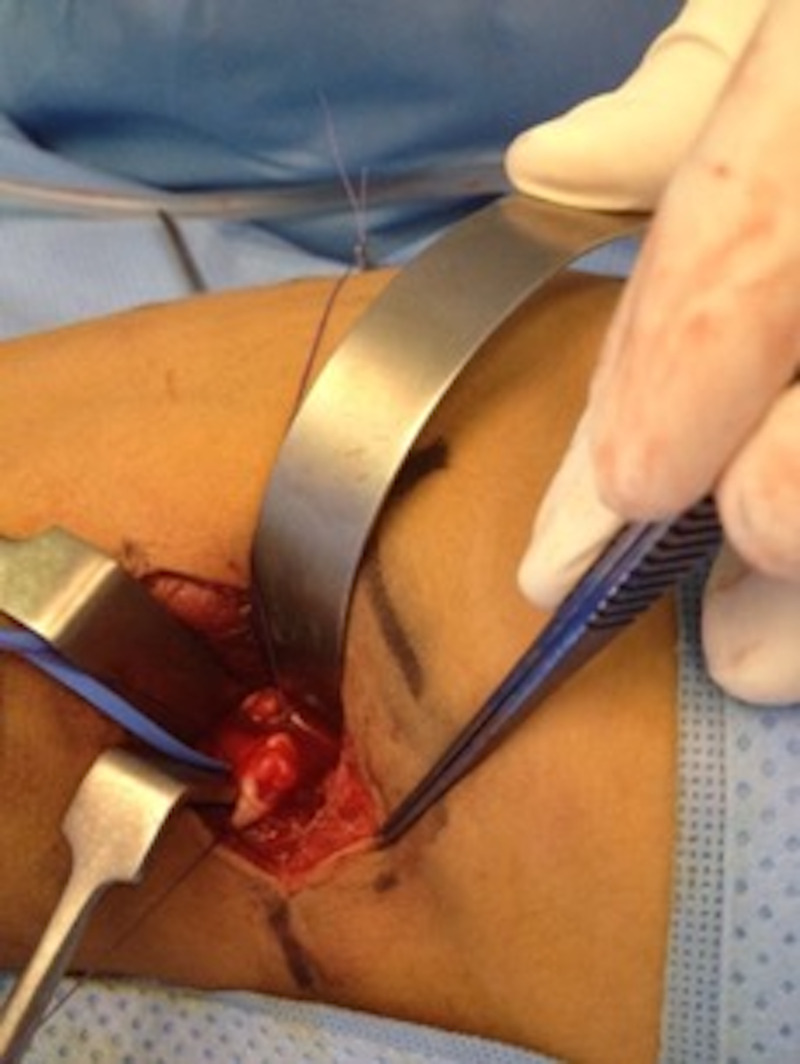
Intraoperative Dissection A large polyp with a smooth surface was seen inside the ureter.

The patient underwent open retroperitoneal surgery with upper ureteral resection and end-to-end anastomosis. Creating a fish-mouth-like opening of the distal end of the ureter was done. Our intraoperative findings included a dilated upper ureter with no aberrant vessels. The fascia around the upper ureter was dissected, and transection was done between the proximal and distal parts of the polyp. Upon opening the ureter, a large ureteral polyp with smooth surfaces was seen (Figure 2). Histopathological findings identified a fibroepithelial polyp.

**Figure 3:**
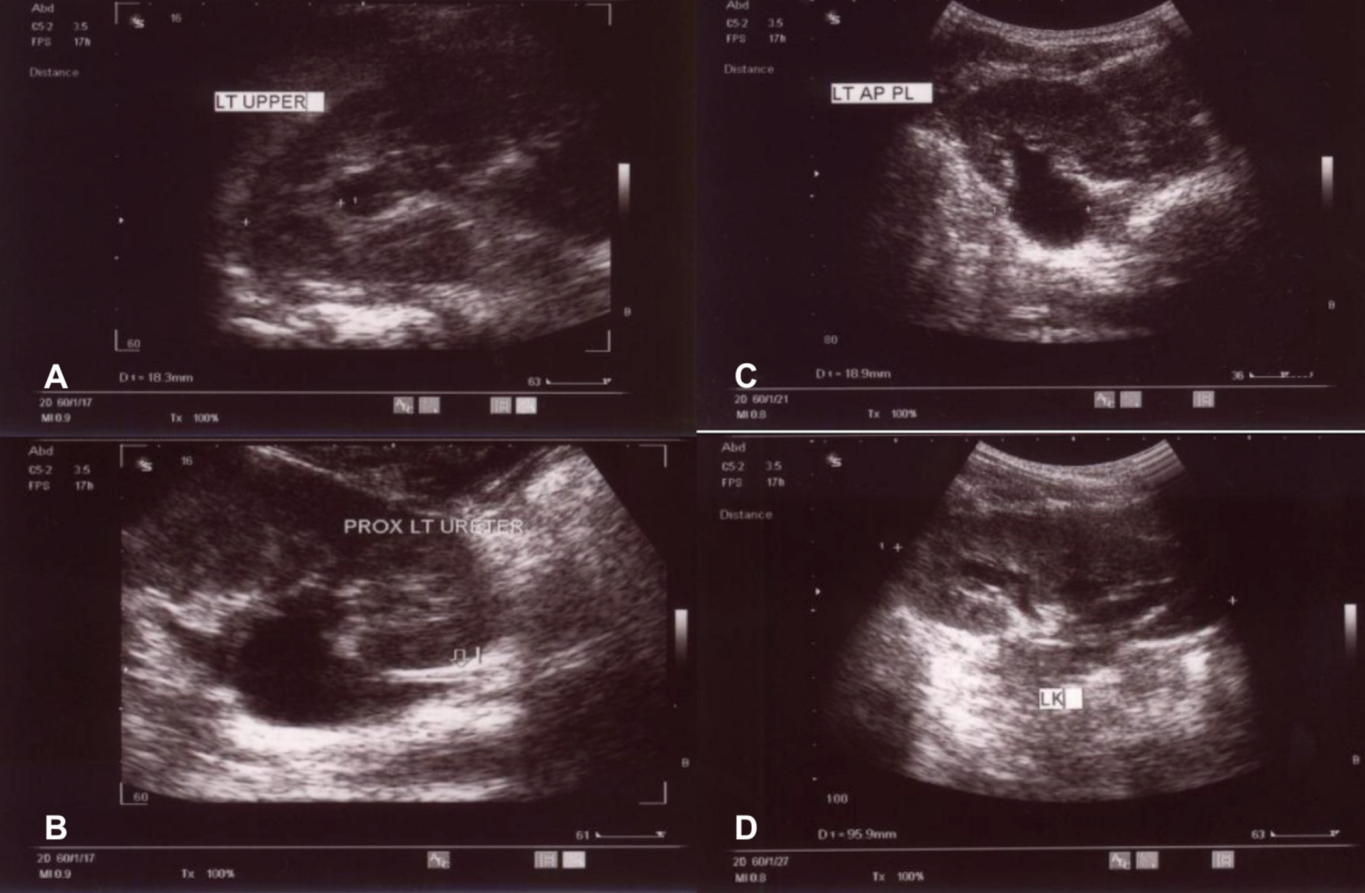
The Postoperative Ultrasound Scan A postoperative ultrasound scan showing normal findings. (A) Left upper ureter. (B) Left proximal ureter. (C) Left renal pelvis. (D) Left kidney.

An ultrasound scan four months following the surgery showed a significant improvement of the upper urinary tract dilation in comparison to the preoperative ultrasound. The anteroposterior diameter of the left renal pelvis was 1.9 cm. The cortical thickness was preserved, measuring 1.8 cm in the upper pole, 1.2 cm in the interpolar area, and 1.4 cm in the lower pole. The upper left ureter was collapsed, as it was on the preoperative studies (Figure 3). Furthermore, a renal function test was normal.

## Discussion

Fibroepithelial polyp of the urinary tract is a rare benign tumor, with a reported incidence of 0.5% [1]. In the literature, there are few cases, and it is mainly reported in adults, making them a rare entity among the pediatric population. Most of these polyps are short, small, and located in the upper ureter and ureteropelvic junction [1]. In contrast, urinary tract polyps are more common in the posterior urethra among the pediatric age group. Patients usually present with symptoms of urinary tract obstruction, while some might also present with flank pain and hematuria resembling symptoms of ureteric stones [4]. Our patient presented with typical flank pain and hematuria.

The diagnosis of a ureteral polyp is done via imaging. The choice of ultrasonography as an imaging modality is the most appropriate course of action as it is readily available, inexpensive, and does not expose the patient to ionizing radiation. It will show hydronephrosis secondary to the obstruction as well as a mildly echogenic non-acoustic shadowing with well-defined margins. Coupling ultrasonography with color Doppler could also show us blood flow in the polyp [4,5]. Wang et al. report the accuracy of an ultrasound scan in detecting ureteral polyps at 62.6% [5]. A positive finding on ultrasonography is an indication for IVP, which will show a filling defect with hydronephrosis secondary to the polyp. Furthermore, a filling defect on IVP prompts the exclusion of other possible causes of ureteral filling defects, such as stones, strictures, clots, and mucosal folds. It is important to note that that the detection rates through IVP can vary from 36% to 100%, and hence other imaging modalities, such as computerized tomography urography (CTU), magnetic resonance urography (MRU), and direct visualization via ureteroscopy, may be used to confirm the diagnosis. Although ureteroscopy is an excellent tool as it offers both diagnostic and therapeutic benefits, its utility is limited in pediatric patients due to the limited working space and relatively large size of the polyp [4-7]. In the presented case, a provisional diagnosis was made using ultrasonography and IVP only as these were sufficient to identify the lesion. Ureteroscopy was not required for this patient, neither in diagnosis nor the treatment, as the lesion was long, and the body size of pediatric patients is not optimal for the procedure.

Treatment of ureteral fibroepithelial polyps can be done endoscopically, laparoscopically, or surgically. The surgeon’s choice depends on the patient’s age, polyp size, number, and location. Previously, polyps within the ureter were treated by nephroureterectomy because of potential malignancy; however as they are benign lesions, a conservative approach is now preferred [4,7]. In children with an obstructive polyp at the ureteropelvic junction, the operation of choice is resection with or without dismembered pyeloplasty. With the advances in endoscopy, ureteroscopic excision has been recommended as a good minimally invasive approach in both adults and children. In fact, many studies report successful experiences with ureteroscopy using electrocautery and holmium laser excision [8,9] However, technical difficulties associated with patient age and polyp size place it at a disadvantage, especially when dealing with pediatric patients. Also, endoscopic procedures are associated with the risk of stenosis from over-vaporization and recurrence due to incomplete resection [9,10]. Laparoscopic surgery has been reported to offer good results as another form of minimally invasive surgery. Laparoscopic exploration provides good visualization of the polyp’s location, size, and anatomical relations. In the pediatric population, laparoscopic surgery is limited due to the lack of adequate working space, and thus, not all approaches provide favorable access to the renal pelvis and ureteropelvic junction [10]. In our case, we performed upper ureteral segmental resection with end-to-end anastomosis because the IVP showed a dilated upper ureter and renal pelvis, a large polyp, and had a patient with a relatively small body; the decision for open surgery was made because of these reasons.

## Conclusions

A fibroepithelial polyp is a rare benign tumor in children and challenging to diagnose and manage. A good outcome is expected if diagnostic workup and choice of management are optimized to the patient. Per our experience, simple ultrasonography coupled with IVP can be sufficient to show and localize the lesion. In our patient, management was successful with upper urinary tract resection and end-to-end anastomosis. Postoperative improvement of ultrasound findings and renal function tests were reassuring.
